# Analysis of the FGF gene family provides insights into aquatic adaptation in cetaceans

**DOI:** 10.1038/srep40233

**Published:** 2017-01-11

**Authors:** Kiwoong Nam, Kyeong Won Lee, Oksung Chung, Hyung-Soon Yim, Sun-Shin Cha, Sae-Won Lee, JeHoon Jun, Yun Sung Cho, Jong Bhak, João Pedro de Magalhães, Jung-Hyun Lee, Jae-Yeon Jeong

**Affiliations:** 1 INRA, UMR 1333 Diversité, Génomes & Interactions Microorganismes–Insectes, 2 place E. Bataillon, 34095 Montpellier, France; 2 Université Montpellier, 2 place E. Bataillon, 34095 Montpellier, France; 3 Marine Biotechnology Research Center, Korea Institute of Ocean Science and Technology, Haeanro 787, Ansan 15627, Republic of Korea; 4 Personal Genomics Institute, Genome Research Foundation, Osong 28160, Republic of Korea; 5 Department of Marine Biotechnology, Korea University of Science and Technology, Daejeon 306-350, Republic of Korea; 6 Department of Chemistry and Nano Science, Ewha Womans University, Seoul, 03760, Republic of Korea; 7 Biomedical Research Institute and IRICT, Seoul National University Hospital, Seoul 110-744, Republic of Korea; 8 The Genomics Institute, Biomedical Engineering Department, UNIST, Ulsan 44919, Republic of Korea; 9 Geromics, Ulsan 44919, Republic of Korea; 10 Institute of Integrative Biology, University of Liverpool, Liverpool L69 7ZB, United Kingdom

## Abstract

Cetacean body structure and physiology exhibit dramatic adaptations to their aquatic environment. Fibroblast growth factors (FGFs) are a family of essential factors that regulate animal development and physiology; however, their role in cetacean evolution is not clearly understood. Here, we sequenced the fin whale genome and analysed FGFs from 8 cetaceans. FGF22, a hair follicle-enriched gene, exhibited pseudogenization, indicating that the function of this gene is no longer necessary in cetaceans that have lost most of their body hair. An evolutionary analysis revealed signatures of positive selection for FGF3 and FGF11, genes related to ear and tooth development and hypoxia, respectively. We found a D203G substitution in cetacean FGF9, which was predicted to affect FGF9 homodimerization, suggesting that this gene plays a role in the acquisition of rigid flippers for efficient manoeuvring. Cetaceans utilize low bone density as a buoyancy control mechanism, but the underlying genes are not known. We found that the expression of FGF23, a gene associated with reduced bone density, is greatly increased in the cetacean liver under hypoxic conditions, thus implicating FGF23 in low bone density in cetaceans. Altogether, our results provide novel insights into the roles of FGFs in cetacean adaptation to the aquatic environment.

Cetaceans (baleen and toothed whales) were derived from extinct, semi-aquatic, deer-like, even-toed ungulates (artiodactyls) approximately 50 million years ago^1^ and have successfully re-populated from terrestrial to aquatic environments. After becoming fully aquatic, the Mysticeti (baleen whales) diverged from the Odontoceti (toothed whale) following the development of keratinous sieves that enabled filter-feeding prior to the onset of the Oligocene Epoch, and subsequently lost teeth completely^2^,^3^.

The anatomical structures, physiology, and metabolism of cetaceans have changed due to various challenges associated with aquatic life. The body shape has been modified to a streamlined form that could reduce fluid resistance^4^. Flukes were developed on their tail for propulsion, hindlimbs were degenerated, and forelimbs were modified into diverse forms of flippers with fused elbow joints that were more suitable for steering than paddling^5^. The hairy fur of their close terrestrial relatives was essentially lost in cetaceans for hydrodynamic reasons^4^, and the bone mineral density was reduced to allow dynamic buoyancy control in deep water^6^. The outer ear pinnae were lost in cetaceans, and the outer ears were functionally replaced by the mandible and the mandibular fat pad, which were better adapted for hearing underwater^4^,^7^. Cetaceans also exhibit various specializations, such as increased oxygen storage capacity, cardiovascular and metabolic adjustments, and increased levels of antioxidants to adapt to oxygen-limited conditions during diving^8^. Despite a thorough understanding of cetacean morphological and physiological alterations, the genetic background underlying these adaptations has only begun to be explored^9^,^10^,^11^.

Fibroblast growth factors (FGFs) are a family of highly conserved growth factors with 22 members in mammals (FGF1 to FGF23; FGF15 and FGF19 are the mouse and human orthologs, respectively). FGFs are further divided into the following three groups: the canonical FGFs, hormone-like FGFs (hFGFs), and intracellular FGFs (iFGFs)^12^. Canonical FGFs function as paracrine factors due to their high affinity for heparin/heparan sulphate proteoglycans (HSPGs) and play essential roles in cell growth and organ development. Members of the hFGF subfamily (FGF19, FGF21, and FGF23) have reduced affinity for HSPGs and function as endocrine factors. The iFGFs (FGF11, FGF12, FGF13, and FGF14) function intracellularly and are independent of FGF receptors^12^.

FGFs have important roles in the development of internal organs; the formation of the limbs, ears, and teeth during embryonic development; and the regulation of bone density, energy homeostasis, and hair growth in adult life^13^,^14^. The diverse functions of FGFs suggest that the evolution of FGF genes in cetaceans may be associated with aquatic adaptations. For example, FGF3 is implicated in ear and tooth development in humans and mice^14^,^15^. FGF10 and FGF20 have essential roles in lung and kidney development, respectively^14^,^16^. Various FGFs, including FGF5, FGF7, FGF10, FGF18, and FGF22, are expressed at high levels in hair follicles and regulate hair growth^13^. Mutations causing the fusion of the elbow and knee joints were discovered in human and murine FGF9^17^,^18^. Furthermore, the emerging roles of FGF19 and FGF21 in the regulation of energy homeostasis and thermogenesis suggest that these factors may be implicated in metabolic modifications in cetaceans^12^,^13^. In addition, the increased activity or the overexpression of FGF23 is associated with decreased bone mineral density, as shown in autosomal dominant hypophosphatemic rickets and tumour-induced osteomalacia in humans^19^. Finally, FGF11, the enigmatic iFGF, was shown to be up-regulated during hypoxia in human endothelial cells and promote capillary-like tube formation when overexpressed^20^. This finding further triggered interest in the involvement of iFGFs in the acquisition of hypoxia tolerance during cetacean evolution.

Although FGF5, a canonical FGF with a suppressive function in hair growth, was shown to be positively selected in cetaceans^21^, the general involvement of the FGF family in the acquisition of cetacean-specific traits is largely unknown. Given the various roles of FGFs in mammalian development and physiology, we hypothesized that molecular adaptations of FGF may be associated with the evolution of the cetaceans. Here, we identified coding sequences of all of the 22 mammalian FGF genes from eight representative cetaceans and analysed the molecular adaptation of FGFs in the course of cetacean evolution.

## Results

### Identification of FGF genes

We sequenced the fin whale (*Balaenoptera physalus*) genome to a 60x average depth of coverage. We also identified or assembled the coding sequences of all 22 mammalian FGF genes (FGF1 to FGF23) from eight representative cetacean species, including three baleen whales (minke whale (*Balaenoptera acutorostrata*), bowhead whale (*Balaena mysticetus*), and fin whale) and five toothed whales (sperm whale (*Physeter macrocephalus*), killer whale (*Orcinus orca*), bottlenose dolphin (*Tursiops truncatus*), finless porpoise (*Neophocaena phocaenoides*), and baiji (*Lipotes vexillifer*, also called the Yangtze river dolphin)), using the newly sequenced fin whale genome, recently published genomes from our labs (minke whale, bowhead whale, and finless porpoise)^22^,^23^ or public databases (killer whale, baiji, sperm whale, and bottlenose dolphin)^24^,^25^.

**Figure 1:**
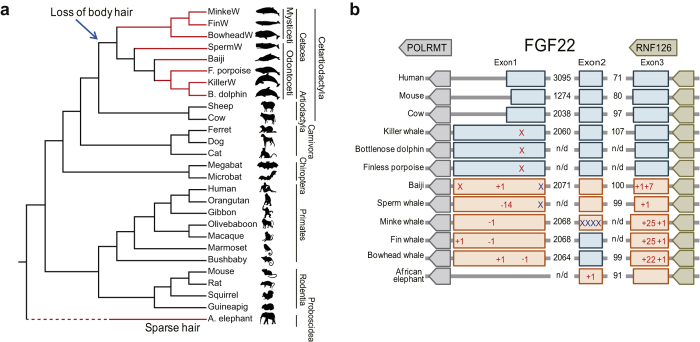
Parallel inactivation of FGF22 in the cetaceans and African elephant. (**a**) The species tree of mammals was drawn based on previous studies^53^,^54^,^55^,^56^ to analyse FGF22. Red lines represent lineages with pseudogenized FGF22. (**b**) Summary of inactivating mutations in FGF22. Nonsense mutations are marked with X in red, and stop codons due to frameshift mutations are marked with X in blue. Indel mutations are expressed with + and −, and exons with frameshift are expressed with red boxes. Animal images were drawn by Oksung Chung and Hana Byun.

To confirm the homology of each FGF family, we constructed a maximum likelihood tree with human and mouse FGFs as references. Each of the FGF subfamilies was clustered into a monophyletic group with high bootstrap values (Supplementary Fig. S1). All cetacean FGFs showed the typical features of FGF, including heparin-binding sites, receptor interaction sites, and localization signals, with the exception of FGF22 (Fig. 1). No nonsense or frameshift mutations were detected in cetacean FGFs, except for FGF3 and FGF22. In cetacean FGF3, a gene with essential roles in ear and tooth development, we found a nonsense mutation that removed a conserved and positively charged Arg-rich motif at the C-terminus (Supplementary Fig. S2). Despite the C-terminal deletion, FGF3 seemed to be functional since no deleterious mutations that disrupt the FGF domain were found in cetaceans, and all of the residues involved in receptor binding or heparin binding were strictly conserved.

Unlike FGF3, independent nonsense or frameshift mutations that destroyed the highly conserved FGF domain were found in FGF22, a gene related with hair follicles, from all cetaceans examined. This finding implies that FGF22 was pseudogenized after the divergence of the toothed and baleen whales (Fig. 1 and Supplementary Figs S3 and S4). The sequences in the vicinity of FGF22 showed a conserved synteny among human, mouse, cow and all of the studied whale species. FGF22 was found between POLRMT and RNF126 without large insertions or deletions in this region (Fig. 1b). FGF22 paralogs were not identified in any other regions. These results exclude the possibility of misassembly due to a lack of callable positions near FGF22 in cetaceans or uncertainty due to the existence of paralogs leading to the misidentification of mutations on FGF22. Furthermore, the lack of FGF22 paralogs indicates that there have been no FGF22 duplication events in the cetacean lineage that could complement the loss of FGF22. Interestingly, we also noticed a loss of FGF22 in African elephant as judged by a complete loss of exon 1 and one nucleotide insertion in exon 2 (Fig. 1 and Supplementary Figs S3 and S4). No deleterious mutations in FGF22 were found in other terrestrial mammals examined here.

### Adaptive evolution of FGF genes

**Figure 2:**
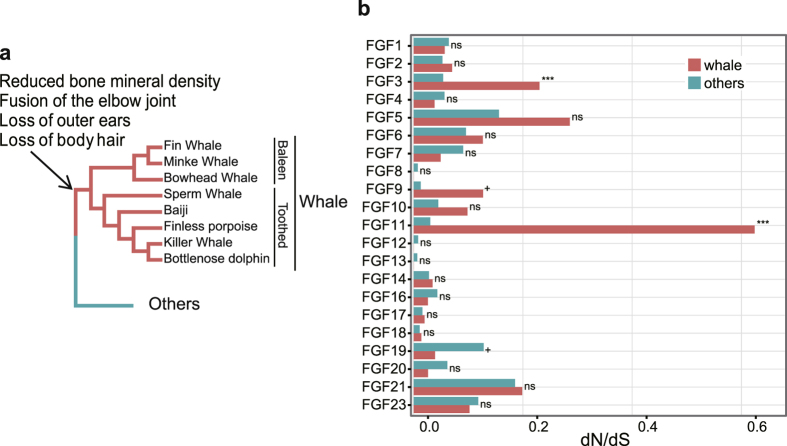
Evolutionary analysis of FGF genes. **(a)** The species tree was drawn based on previous studies^53^,^54^,^55^,^56^. **(b)** The *d*_*N*_*/d*_*S*_ ratio of whale branches (orange) and the other mammalian branches (turquoise) for each FGF gene. The ns, +, and *** denotes the significance level with p > 0.1, p < 0.1, and p < 0.001, respectively.

The strength of the selective pressure acting on FGF genes was analysed using the nonsynonymous to synonymous substitution rate ratio (*d*_*N*_*/d*_*S*_, also termed ω) based on the species tree shown in Fig. 2a 26. First, we contrasted the strength of selection between whales and other mammals using the branch model^27^. The average *d*_*N*_*/d*_*S*_ ratios of the whale lineages and those of the remaining lineages were calculated under a two ratio model that allows different *d*_*N*_*/d*_*S*_ ratios between whale branches and the other branches. The mean *d*_*N*_*/d*_*S*_ ratios ranged from 0.0001 (FGF8, FGF12, and FGF13) to 0.6242 (FGF11) in whales and 0.0066 (FGF13) to 0.1855 (FGF21) in the other mammalian species we investigated (Supplementary Table S1). The *d*_*N*_*/d*_*S*_ ratio was significantly correlated between whales and other mammals (spearman’s rho = 0.61, p-value = 0.003081). We also performed a likelihood ratio test if the *d*_*N*_*/d*_*S*_ ratio was significantly different between whale branches and the other branches for each of FGF genes. None of the FGF genes had a significantly different *d*_*N*_*/d*_*S*_ ratio between whales and other mammals, with two exceptions. FGF3 and FGF11 had a significantly higher *d*_*N*_*/d*_*S*_ ratio in whales (adjusted p-value = 0.00031 and <2.2 × 10^−16^, respectively) than in other mammals (Fig. 2b and Supplementary Table S1). This result implies that strong purifying selection has acted on FGFs to a comparable extent between whales and other mammals, except for at FGF3 and FGF11; these genes have experienced relaxed purifying selection or substantial positive selection in the whale lineage.

To test the presence of positive selection in the whale lineage, we used branch site model^28^. With this model, the phylogenetic tree shown in Fig. 2a was divided into foreground branches, which were composed of ancestral and terminal whale branches and in which the proportion of codons with *d*_N_/*d*_S_ > 1 was estimated, and background branches, which were composed of the other branches and in which no codon was allowed to have dN/dS > 1. We found that no FGF genes showed a significant signature of positive selection in the whale lineage (Likelihood ratio test, Supplementary Table S2). One assumption of the branch site model is that positive selection has not occurred in the non-whale mammals, and there is no reason to believe that this assumption is supported *a priori*. Thus, we used an alternative approach. To infer signatures of positive selection in whales, we analysed only the whale branches by reconstructing ancestral whale sequences using a maximum likelihood approach and by generating multiple sequence alignments composed of both the inferred ancestral whale sequences and the extant whale sequences. Then, we used the site model^29^ to test for positive selection (Table 1). For FGF3, the proportion of positively selected codons was greater than zero (corresponding to p2 at M2 in Table 1, 0.644%; raw p-value = 0.0026, and adjusted p-value = 0.055). This result implies that FGF3 may be under positive selection.

To detect possible positive selection that has occurred along whale branches with a resolution of a single amino acid, we used the Mixed Effects Model of Evolution (MEME)^30^ based on the phylogenetic tree and multiple sequence alignments of extant whales and the inferred ancestral sequences. The MEME estimates the rates of non-synonymous substitution and synonymous substitution for each codon. The difference between these two ratios was tested using a likelihood ratio test and any branch under positive selection was evaluated with an empirical Bayes factor. Of all the FGF genes, only FGF3 and FGF11 showed a significant signal of positive selection (Supplementary Figs S5 and S6). In FGF3, I85L was shown to be under positive selection (p = 0.0235), with the strongest signal observed in minke whale. For FGF11, a significant positive selection was observed at S234T (p = 0.0179), with the strongest signal in baiji. Marginal significance was also observed at 237 of FGF11 (p = 0.0728). This result suggests that positive selection has been acting on FGF3 and FGF11, genes related to ear and tooth development and hypoxia, respectively.

### Changes in FGF genes and the emergence of whales

We investigated how phenotypes and physiological aspects of extant whales were generated after diverging from a sister taxa of whales. Among all FGF genes investigated, we identified 13 amino acid replacements in eight FGF genes that occurred after diverging from a sister taxa of whales but prior to the divergence between Mysticeti and Odontoceti (Table 2). The PolyPhen-2^31^ analysis showed that three replacements were predicted to have a drastic function-altering effect, expressed as probably or possibly damaging, and ten replacements were predicted to be neutral. The rate of function-altering substitutions was 162% greater along the ancestral branches of whales than along the other branches (Table 3). However, the neutral substitution rate differed by only 3.8%. We also used two other algorithms, SIFT^32^ and PROVEAN^33^, to predict whether the observed mutations had a drastic function-altering effect. The results from these two algorithms showed the same pattern as that from PolyPhen-2 (Table 3). If we assume that the neutral mutations may actually have had slightly deleterious effects, this comparable neutral substitution rate suggests that the strength of purifying selection was largely the same between lineages of ancestral whales and the rest of the mammalian lineage. Thus, higher rates of function-altering substitutions in ancestral whales were likely to be due to stronger positive selection on FGF genes.

**Figure 3:**
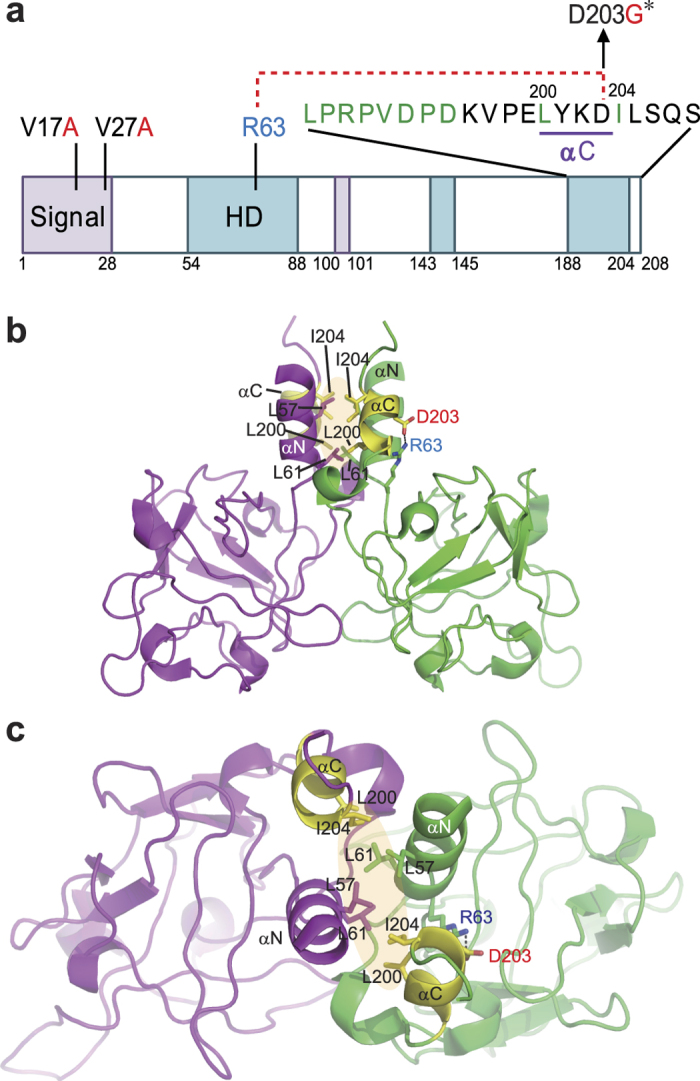
The D203G substitution in cetacean FGF9 might affect the homodimerization of FGF9. (**a**) Schematic drawing of mammalian FGF9. Cetacean-specific substitutions are marked in red, and residues involved in the homodimerization of FGF9 are marked in green. The C-terminal α helix is underlined, and the salt bridge between Arg63 and Asp203 is shown as a red broken line. Bipartite signal sequences and homodimerization domains are marked with purple and blue boxes. HD, homodimerization domain. (**b,c**) Ribbon drawing of the dimeric human FGF9 with each monomer in different colours. Side (**b**) and top (**c**) views. Residues involved in homodimerization (Leu57, Leu61, Leu200 and Ile204) and stabilization of the C-terminal α helix (Arg63 and Asp203) are shown as sticks. The black dotted line indicates the salt bridge between Arg63 and Asp203. The C-terminal α helix and Ile204 are shown in yellow, and the transparent orange oval represents the hydrophobic core at the dimeric interface. αN, N-terminal α helix; αC, C-terminal α helix. *Function-altering change predicted by PolyPhen-2, SIFT, and PROVEAN.

The replacement of D203G in FGF9 was predicted to have a damaging effect by all algorithms used (Table 2). Gly203 resides in the short C-terminal helix that is composed of residues 200–203 (Fig. 3a). According to the crystal structure of dimeric human FGF9^34^, the negatively charged side chain of Asp203 forms a salt bridge with the guanidinium group of Arg63 (Fig. 3b,c). The favourable ionic interaction that is mediated by Asp203 cannot occur in the cetacean FGF9 harbouring the D203G substitution. Given that the stability of helices is affected by the side chain-mediated interactions^35^, the D203G substitution is highly likely to destabilize the short C-terminal helix. Leu200 in the helix and Ile204, the adjacent residue, form a hydrophobic core at the dimeric interface. The helical conformation of residues 200–203 is important to correctly position Leu200 and Ile204 at the dimeric interface. Therefore, the cetacean FGF9 with the D203G substitution might be defective in homodimerization.

### Induction of FGF11 in the brain and heart of the Cetacea during hypoxia

**Figure 4:**
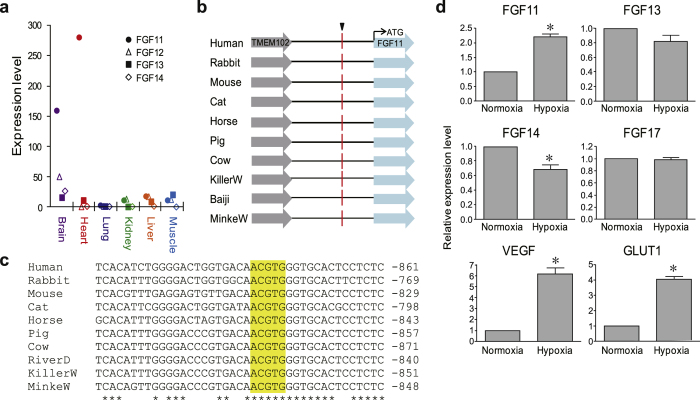
Inducible expression of FGF11 in the cetacean brain and heart upon hypoxia. (**a**) Increased levels of FGF11 transcripts in the brain and heart of a minke whale revealed by RNA-seq analysis. (**b**,**c)** A conserved HRE in FGF11 promoter marked with red line (**b**) and highlighted in yellow (**c**). (**d**) Induction of FGF11 upon hypoxia. SH-SY5Y cells were incubated with normoxia or hypoxia (1% O_2_) for 18 h, and RNA levels were determined using quantitative RT-PCR (n = 4, mean ± SEM, *p < 0.05).

To understand the role of FGF11 in the evolution of the cetaceans, we examined the expression of FGF11 in various tissues using RNA-seq data from a minke whale and five bowhead whales^22^,^23^,^36^. The expression levels of iFGFs were higher in the brain than in other organs (Supplementary Tables S3 and S4), consistent with results from mouse^37^. Interestingly, the expression of FGF11 was markedly increased in the brain and heart of the minke whale (Fig. 4a and Supplementary Table S3), but was not detected in the bowhead whales (Supplementary Table S4). The minke whale sample was obtained as bycatch, with drowning being the main cause of mammalian death from bycatch, while the bowhead whales were caught by hunting^23^,^36^. Thus, the differential levels of FGF11 between minke and bowhead whales suggest that FGF11 was induced by hypoxia in the minke whale. To examine this possibility, we searched for the presence of Hypoxia-Inducible Factor-1 binding site, the master regulator of hypoxia response, within FGF11 promoters from representative mammals. A consensus hypoxia response element (HRE)^38^, ACGTG, was identified as being conserved in all the mammals examined, thus implying that FGF11 could be a direct target of Hypoxia-Inducible Factor-1 (Fig. 4b,c).

To determine if FGF11 is induced by hypoxia in nervous tissues, we analysed gene expression profiles in SH-SY5Y human neuroblastoma cells. Under hypoxic conditions, the expression of FGF11 was significantly increased, along with Vascular Endothelial Growth Factor and Glucose Transporter 1, well-known markers for hypoxia (Fig. 4d), suggesting that the increased expression of FGF11 in the minke whale reflects the hypoxic status of the animal. In contrast to FGF11, expression of FGF14 was decreased and expression of FGF13 and FGF17 were unchanged by hypoxia, implying that the induction of FGF11 during hypoxia is not a general feature that is shared with other FGFs. Taken together, our results provide evidence that FGF11 is induced under hypoxic conditions in the brain and the heart of cetaceans, the most sensitive tissues to hypoxic damages.

### Induction of FGF23 in the liver during hypoxia and the acquisition of HREs in the promoter of cetacean FGF23

**Figure 5:**
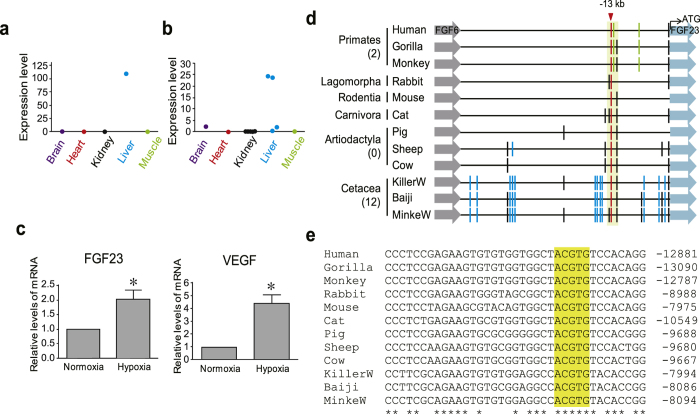
Expression of FGF23 in the cetacean liver and increased number of cetacean-specific HREs in the FGF23 promoter. (**a**,**b**) RNA-seq analysis showed high levels of FGF23 transcripts in the livers from a minke whale (**a**) and two bowhead whales (**b**). (**c**) Induction of FGF23 upon hypoxia. HepG2 cells were incubated in normoxia or 1% O_2_ for 18 h, and mRNA levels for FGF23 and VEGF were investigated using quantitative RT-PCR (n = 3, mean ± SEM, *p < 0.05). (**d**) HREs conserved in at least three species are shown. A conserved HRE across all the mammalian species is shown about −13 kb region in the FGF23 promoter (red line). HREs specific to the Primates and the Cetacea are marked with green and blue lines, respectively. The number of the specific HREs are shown in parentheses. (**e**) Multiple nucleotide sequence alignment of the promoter region encompassing the conserved HRE is highlighted in yellow.

FGF23 is one of the main regulators of bone mineral density and is mainly produced in bone tissue^19^. Unexpectedly, we found a dramatic increase of FGF23 in the liver of minke whale^23^ (Fig. 5a and Supplementary Table S3) and two bowhead whales (Fig. 5b and Supplementary Table S4)^22^,^36^, with a greater increase in the minke whale. Considering that FGF23 is not expressed in normal liver from other mammals^19^, we investigated if the expression of FGF23 in cetacean liver was the result of adaption to unique environmental conditions. As the minke whale was obtained as bycatch, whereas the bowhead whales were obtained through hunting, the differential gene expression of FGF23 suggests differences in hypoxic status. In human HepG2 hepatoma cells, we observed that FGF23 was expressed under hypoxia (Fig. 5c), implying that hypoxia can induce the expression of FGF23 in the liver in mammals.

In the promoter regions, we identified a conserved HRE approximately 13 kb upstream of FGF23 in all the mammals examined (Fig. 5d,e). Interestingly, the number of cetacean specific HREs (twelve) was much higher than that of primates (two) or Artiodactyla (zero) (Fig. 5d). The increased number of HREs in cetaceans suggests the possibility that cetaceans have evolved to produce FGF23 more efficiently under hypoxic conditions during diving.

Although it is well known that cetaceans experienced dramatic bone density reductions during their habitat transition from shallow to deep water resulting in dynamic buoyancy control^6^, the underlying mechanism is not known. Because the overexpression of FGF23 decreases bone density^19^, the acquisition of cetacean-specific HREs may have contributed to the development of the extremely low bone density in cetaceans by facilitating FGF23 expression in the liver.

## Discussion

Here, we studied the molecular adaptation of FGF genes in the course of cetacean evolution through evolutionary analysis, homology modelling, and expression analysis. We found significant signatures of positive selection in FGF3 and FGF11, a potentially function-altering mutation in FGF9 in ancestral whales, acquisition of cetacean-specific HREs in the promoter region of FGF23, and functional loss of FGF22 in all cetaceans. These evolutionary events appear to be closely correlated with the advent of morphology and physiology of extant cetaceans.

We observed the functional loss of FGF22 in all cetaceans and African elephant (Fig. 1). Each of the FGF subfamilies was derived from an FGF13-like ancestral gene through gene duplications prior to the evolution of vertebrates^14^, and each subfamily further expanded to multiple members through two rounds of whole genome duplication during the evolution of early vertebrates^14^,^39^. The 22 members of the mammalian FGF family were established after the loss of FGF24 in the ancestry of tetrapods^39^. Although the loss of FGF11, FGF17, and FGF21 were identified in some avian lineages, inactivation of FGF22 has not thus far been reported in other tetrapods^39^.

FGF22 is preferentially expressed in the inner root sheath of the hair follicle^40^, but its role in hair development or maintenance is not yet clearly understood^41^. The independent, damaging mutations in baleen and toothed whales suggest that FGF22 was no longer necessary when the common ancestor of the cetaceans lost its body hair (Fig. 1)^9^. Interestingly, we also found damaging mutations in African elephant, an animal with very sparse hair (Fig. 1). The African elephant has lost most of its body hair in response to the high demand for efficient heat transfer because it has the largest body volume to surface area ratio among terrestrial mammals and lives in hot environments^42^. The parallel loss of FGF22 in cetaceans and African elephant indicates the importance of FGF22 in the maintenance or control of the density of hair in mammals. It would be interesting to examine whether FGF22 is maintained in other mammals, e.g., hippopotamus, that have reduced or no skin hairs.

We also observed a significant signature of positive selection on FGF3 in the cetacean lineage (Table 1). FGF3 is known to play important roles in the formation of the ears and teeth in humans and mice^15^,^43^. Cetaceans have undergone major changes in the ear and tooth including loss of the external ears, development of mandibular fat body, isolation of the tympanoperiotic complex from the skull, and loss of occlusion of the teeth^7^,^44^. Thus, the positive selection on FGF3 is potentially associated with changes in these morphological traits^3^,^4^,^7^,^44^.

We also found a cetacean-specific loss of positively charged Arg-rich motif in FGF3 (Supplementary Fig. S2). Because all the essential components of the gene, such as signal sequence, receptor binding sites, and heparin-binding sites, are strictly conserved, lack of the Arg-rich motif is likely to modify rather than abrogate the function of FGF3. Given that FGF3 functions as a paracrine factor by binding to negatively charged HSPGs in the extracellular matrix, and Arg binds heparin approximately 2.5-fold more tightly than does Lys^45^, the Arg-rich motif might be involved in heparan sulphate binding and regulating local concentrations of FGF3 in addition to the canonical heparin binding sites within the FGF domain. Similarly, it was recently shown that modulation of FGF3 dosage is related to the evolution of mammalian dentition^46^. It would be intriguing to determine if the specific loss of the Arg-rich motif affects the heparin binding affinity of FGF3.

In cetacean FGF9, three amino acids were replaced in the common ancestor of the cetaceans (Table 2). Among these, the D203G substitution was predicted to alter the function of FGF9. Homology modelling further suggested a defective homodimerization of FGF9 because of this substitution (Fig. 3). Interestingly, it is already known that mutations affecting homodimerization of FGF9 are associated with the fusion of the elbow and knee joints in mice and humans^17^,^18^. The modification of the cetacean forelimb into an inflexible flipper, resulting in more efficient locomotion, occurred prior to the last common ancestor of extant cetaceans^9^; however, the genetic mutation underlying this morphological change has not yet been identified. Our results, together with previous reports, suggest that the D203G substitution in FGF9 is responsible for the morphological change in the cetacean joint.

Hypoxia tolerance is one of the main characteristics of the cetaceans^8^. Recently, evidence of adaptive evolution was found in genes involved in transporting oxygen to the blood and muscle (haemoglobin-α and β, myoglobin) and the regulation of vasoconstriction (endothelin-1, -2, and -3, endothelin receptor type A and B, adrenergic receptor α-1D, and arginine vasopressin)^10^,^11^. Here, we also detected a signature of positive selection in FGF11 along the Cetacea (Supplementary Figs S5 and S6), together with a cetacean-specific function-altering amino acid replacements identified by Polyphen-2 (Table 2). Through expression and promoter analyses, we showed an increased expression of FGF11 in the brain and heart in cetaceans and provided compelling evidence of FGF11 induction during hypoxia (Fig. 4). FGF11 belongs to the iFGF subfamily, which is known to be highly expressed in the nervous system^37^. Other members of the iFGF subfamily were reported to bind voltage-gated sodium channels^47^ and display altered phenotypes in the nervous system when deleted^14^. In contrast, gene expression levels of FGF11 in the brain were relatively low, and the function of FGF11 remains largely unknown^14^. In a previous report, we found the inducible expression of FGF11 in human umbilical vein endothelial cells by hypoxia^20^. The expression of FGF11 increased capillary-like tube formation in these cells, suggesting a protective role of FGF11 during hypoxia. Increased hypoxia tolerance is one of the main characteristics of the cetaceans, but underlying molecular mechanisms are not yet clearly understood. Our results provide a clue to the function of FGF11 during hypoxia and suggest that molecular adaptation of FGF11 may have played a role in the evolution of the Cetacea by providing hypoxia tolerance in the brain and heart, two pivotal organs to protect during prolonged hypoxic dives.

We also discovered a wide range of gene expression levels of FGF23 in the livers from minke and bowhead whales (Fig. 5). FGF23 is a bone-derived hormone that acts on the kidney to inhibit phosphate reabsorption and vitamin D3 synthesis. FGF23 is induced by increased levels of blood phosphate and vitamin D3, but mechanisms underlying aberrant expression in ectopic tissues during pathological conditions have not yet been clearly understood^19^. Although Bhattacharyya *et al*. proposed that hypoxia causes FGF23 induction in osteoblast, HRE in the FGF23 promoter was not identified because only 5 kb of the upstream region was investigated^19^. By analysing the entire upstream sequence between FGF23 and the nearest gene (~50 kb), we identified a conserved HRE approximately 13 kb upstream of FGF23 and proved inducible expression of FGF23 in a human hepatic cell line. These results explain an important response to hypoxia in the cetacean liver and the general regulatory mechanism of FGF23 expression in other mammals during pathologic conditions, such as tumour-induced osteomalacia and chronic kidney diseases^19^.

During the first quarter of cetacean evolution, cetacean bone density was transformed dramatically from the typical terrestrial form to osteopetrosis and finally to osteoporosis, depending on the habitat^6^. Archaeological evidence shows that early archaic cetaceans living in shallow water displayed high bone density that provided static buoyancy control (ballast), while late ancient cetaceans living in deep water exhibited extremely low bone density that allowed for dynamic buoyancy control^6^. Unlike other morphological changes, such as flippers and flukes that are formed during embryonic stages, maintaining low levels of bone density requires lifetime regulation. Because the overexpression of FGF23 is linked with low bone density^19^, the induction of FGF23 by hypoxia, which occurs during diving, provides a reasonable explanation for the underlying mechanism of lifetime adjustment and maintenance of low bone density in animals living in deep water.

Surprisingly, we found that cetaceans had a higher number of HREs in the FGF23 promoter than did the other terrestrial mammalian orders studied. This result raises the possibility that cetaceans evolved to produce FGF23 more efficiently in the liver during their transition from shallow water to deep water, concomitant with the increased frequency and intensity of hypoxia during deep and prolonged diving. Although further experiments are required to characterize the contribution of individual HREs to the expression of FGF23, our findings advance the understanding of the molecular basis of cetacean bone evolution during the course of aquatic adaptation.

We also identified cetacean-specific mutations in FGF10 (D43G), FGF20 (R46L), FGF19 (L113S), and FGF21 (E119Q) that were predicted to affect the function of these genes (Table 2). It would be intriguing to determine if these changes are linked with the evolution of the lung (FGF10), or kidney (FGF20), or with the metabolic changes (FGF19 and FGF21) required for aquatic life in cetaceans.

Taken together, our findings provide various novel insights into the roles of FGFs in the aquatic adaptations exhibited by cetaceans. In addition, the whole genome sequences of the fin whale will be an important resource for future studies of cetacean evolution, biology, and genetics.

## Methods

### Fin whale sequencing and identification of FGF coding sequences

Genomic DNA was extracted from a fin whale that had been accidentally killed and investigated by the Korean maritime police. The whole genome was sequenced to 60x average read depth using Illumina HiSeq2000 with a 400 bp insert library at Theragen BiO Institute (TBI); sequence data have been deposited in GenBank (SRA346104). The genome sequence of the fin whale and the genome sequences of one fin whale and one finless porpoise that had been sequenced previously^23^ were constructed using the consensus method. The consensus method utilizes the genome sequences of the most closely related species and maps short reads to these sequences resulting in a consensus genome sequence. In this study, short reads from the three cetaceans were mapped to the genome sequences of their closest sister species (Supplementary Table 5). The mapping was conducted using BWA-MEM^48^ with the default options, and rmdup, implemented in SAMtools 0.1.19^49^, was used to remove potential PCR duplicates among the short reads. SAMtools 0.1.19 was used for variant calling with the “-d 5” option. The consensus sequences were obtained by replacing nucleotides from the reference genome sequences with the variants. The genome sequence of the bowhead whale was obtained from The Bowhead Whale Genome Resource^22^ (http://www.bowhead-whale.org/).

We analysed all of the mammalian FGF genes (FGF1 to FGF23) from 24 terrestrial mammals and 8 cetaceans; a list of the species is provided in Supplementary Table 5. Using human FGF coding sequences obtained from the UniGene database (http://www.ncbi.nlm.nih.gov/UniGene) as queries, the FGFs of the terrestrial mammals and 5 cetaceans (minke whale, bottlenose dolphin, killer whale, baiji and sperm whale) were obtained from the GenBank or Ensembl (http://www.ensembl.org) database through BLAST search. The coding sequences of the FGF genes from the fin whale, finless porpoise, and bowhead whale were extracted from their genome sequences.

Minke whale FGF22 genomic regions were amplified using the GC-RICH PCR system (Roche, Basel, Switzerland) with primers FGF22-E1-F, 5′-CACCCACTGTGGCTCTGG-3′ and FGF22-E1-R, 5′-GATCCACACACAGGAAGAAGTG-3′ for exon 1; and FGF22-E2-F, 5′-AGGCGTCTTCTTCGAGTTGCGC-3′ and FGF22-E3-R, 5′-TCAGGAGACCAGGACGGGCAG-3′ for a region spanning exons 2 and 3. The PCR was performed with 1 M GC-RICH resolution solution provided from the supplier. The PCR conditions were as follows: 95 °C for 3 min, followed by 25 cycles of 95 °C for 30 sec, 60 °C for 30 sec and 72 °C for 1 min +5 sec/cycle, and a final extension of 72 °C for 7 min. PCR products were cloned into pGEM-T Easy vector (Promega, USA) and sequenced. A list of the species, sequence identification numbers, and gene identification methods used in this study are summarized in Supplementary Tables S5 and S6.

### Phylogenetic tree construction for FGF genes

The coding sequences were aligned using ClustalW in MEGA6, and a phylogenetic tree was constructed using the Maximum Likelihood method based on the General Time Reversible model^50^, implemented in MEGA6^51^. The initial tree for the heuristic search was obtained automatically by applying the Neighbour-Joining and BioNJ algorithms to a matrix of pairwise distances estimated using the Maximum Composite Likelihood (MCL) approach; the topology with the superior log likelihood value was selected. We performed 100 times of bootstrap replications.

### Molecular evolutionary and comparative analyses of FGF genes

We used the PRANK program^52^ for the multiple sequence alignment of the coding sequences of the FGF genes with the empirical codon model option. The CODEML program in PAML 4.5^26^ was used to estimate *d*_*N*_/*d*_*S*_ ratios based on the framework of maximum likelihood. The guide tree was drawn based on previous studies^53^,^54^,^55^,^56^. We used the branch model to calculate the *d*_*N*_/*d*_*S*_ ratio for whales and the other mammals. With the two-ratio model^27^, the *d*_N_/*d*_S_ ratios for whales and the other mammals were estimated separately, and the corresponding likelihoods were calculated. To test if the *d*_N_/*d*_S_ ratios were significantly different between whales and the other mammals, we calculated the mean *d*_N_/*d*_S_ ratio from the entire phylogenetic tree (one ratio model) and the likelihood was calculated. Then, a log likelihood ratio test was performed to test if *d*_*N*_/*d*_*S*_ ratios were significantly different between whales and the other mammals (df = 1), with an FDR correction for multiple tests.

To further test for signatures of positive selection, we used a branch-site model^28^. For each analysis, whale branches composed of ancestral whale branches and terminal whale branches were defined as foreground branches and the remaining branches were defined as background branches. In the selection model, the proportion of codons that had *d*_*N*_/*d*_*S*_ ratios greater than 1 in the foreground branch was estimated to be greater than zero. In the neutral model, no codon was allowed to have a *d*_*N*_/*d*_*S*_ ratio greater than 1. Then, a likelihood ratio test was performed using the difference in likelihoods between the selection and neutral models to test if the selection model had greater explanatory power than the neutral model to describe the empirical data. The positive selection model was considered unsupported if twice the difference in likelihood did not exceed 3.84, which is the recommended critical value for a significance level of 5% (Yang Z; http://abacus.gene.ucl.ac.uk/software/pamlDOC.pdf).

We also used a site model to identify positive selection^29^. The ancestral codon sequences of whales were inferred using the CODEML program implemented in the PAML package^26^. Alignments were made with the extant whale sequences and the inferred ancestral whale sequence and signatures of positive selection were identified from all branches using the site model^29^. The selection model (M2) in which local *d*_N_/*d*_S_ ratio was allowed to be greater than 1 was compared with the neutral model (M1) in which local *d*_N_/*d*_S_ ratio was not allowed to be greater than 1. In M2, an alignment was categorized into three groups. In group 0, 1, and 2, the *d*_N_/*d*_S_ was lower than 1, equal to 1, and greater than 1, respectively. And the proportions of codons at group 0, 1, and 2 were calculated together with the estimated *d*_N_/*d*_S_ ratios for group 0 and group 2. In M1, there were only group 0 and group 1, thus there was no codon with *d*_N_/*d*_S_ greater than 1. A likelihood ratio test (df = 2) and FDR correction for multiple tests were performed to test for statistical significances.

To detect signatures of positive selection on each codon, we used the MEME algorithms^30^, implemented in the datamonkey web application^57^, together with the whale phylogenetic tree shown in Fig. 2a and the multiple sequence alignments for extant whales and their ancestors. The non-synonymous substitution rate (β) and synonymous substitution rate (α) at each codon of an alignment was estimated and the difference between α and β was tested by likelihood ratio test. And Empirical Bayes factors were calculated to estimate the relative likelihood of positive selection at each branch.

### Prediction of the functional effects of ancestral cetacean mutations

Function altering amino acid changes were predicted using PolyPhen-2^31^, PROVEAN v1.1^33^, and SIFT^32^, with the default cutoff values, and human protein sequences were used as queries for the programs. First, amino acid replacements were identified using the multiple sequence alignments of each FGF gene. Second, we classified these replacements into the following two groups: amino acid replacements that were generated in the whale lineages and amino acid replacements arising in the other lineages. Third, we run the web applications of PolyPhen-2 ((http://genetics.bwh.harvard.edu/pph2/) and PROVEAN v1.1 and SIFT (http://provean.jcvi.org/). The predictions ‘possibly damaging’ or ‘probably damaging’ by PolyPhen-2 were regarded as a function-altering replacements. The predictions ‘Deleterious’ and ‘Damaging’ by PROVEAN v1.1 and SIFT, respectively, were also regarded as function-altering replacement.

### Promoter analysis

Promoter regions of FGF11 and FGF23 were obtained from NCBI and analysed using the Kalign tool (http://www.ebi.ac.uk/Tools/msa/kalign/). GenBank accession numbers are listed in Supplementary Tables S7 and S8.

### Cell culture, hypoxia treatment, and quantitative RT-PCR

HepG2 and SH-SY5Y cells lines were obtained from the American Type Culture Collection and Korean Cell Line Bank, respectively. For hypoxia treatment, cells were incubated in a Modular Incubator Chamber (Billups-Rothenberg Inc., CA, USA) filled with 1% oxygen, 5% CO_2_, and 94% nitrogen for 18 h. Total RNA was prepared, and cDNA was synthesized using reverse transcriptase. Quantitative PCR was performed using Thunderbird SYBR qPCR Mix reagent (TOYOBO, Japan) and a CFX Connect Real-Time PCR system (BioRad, CA, USA). ACTB was used as an endogenous control. Primer sequences can be found in Supplementary Table S9.

### RNA-seq analysis

RNA sequences were obtained from previously published minke^23^ and a bowhead whale^22^ data, and from four bowhead whales published by Seim *et al*.^36^ and mapped against their own reference genome using the TopHat2 with default options^58^. The GFF3 annotation files were downloaded from NCBI. The number of reads that were mapped against coding sequences of FGF genes was counted using HTSeq-0.6.1^59^ and normalized by Trimmed Mean of M-values (TMM) using edgeR^60^.

## Additional Information

**How to cite this article:** Nam, K. *et al*. Analysis of the FGF gene family provides insights into aquatic adaptation in cetaceans. *Sci. Rep.* **7**, 40233; doi: 10.1038/srep40233 (2017).

**Publisher's note:** Springer Nature remains neutral with regard to jurisdictional claims in published maps and institutional affiliations.

## Supplementary Material

Supplementary Information

Supplementary Dataset 1

## Figures and Tables

**Table 1:** Positive selection on FGF genes.

Gene	Length	M2						M1				diff	p value	adjusted p value
		*p*_0_	*p*_1_	*p*_2_	ω_0_	ω_2_	L	*p*_0_	*p*_1_	ω_0_	L			
FGF1	459	0.993	0	0.006	0.019	20.467	−728.86	0.956	0.043	0	−732.42	7.10	0.0286	0.3011
FGF2	453	0.990	0	0.009	0	8.096	−688.43	0.960	0.039	0	−689.63	2.39	0.3017	1
FGF3	480	0.841	0.151	0.006	0.024	36.996	−919.43	0.843	0.156	0.020	−925.36	11.86	0.0026	0.0556
FGF4	588	1	0	0	0.038	1	−927.87	1	1E-005	0.038	−927.87	0.00	0.9996	1
FGF5	747	0.812	0.078	0.108	0.165	1	−1296.74	0.812	0.187	0.165	−1296.74	0	1	1
FGF6	618	0.871	0.108	0.019	0	1	−1074.37	0.871	0.128	0	−1074.37	0	1	1
FGF7	579	0.991	0	0.008	0	16.169	−813.34	0.925	0.074	0	−813.45	0.21	0.8980	1
FGF8	408	1	0	0	0	6.838	−624.81	1	1E-005	0	−624.81	0.00	0.9993	1
FGF9	624	1	0	0	0.152	1	−941.66	1	1E-005	0.152	−941.66	0.00	0.9999	1
FGF10	588	1	0	0	0.085	1	−936.85	1	1E-005	0.085	−936.85	0.00	0.9999	1
FGF11	567	0.544	0.317	0.137	0.254	1	−914.52	0.544	0.455	0.254	−914.52	0	1	1
FGF12	720	1	0	0	0	1	−1063.87	1	1E-005	0	−1063.87	0.00	0.9996	1
FGF13	675	1	0	0	0	1	−990.05	1	1E-005	0	−990.05	0.00	0.9996	1
FGF14	738	1	0	0	0.029	1	−1130.69	1	1E-005	0.029	−1130.69	0.00	0.9999	1
FGF16	621	0.994	0	0.005	0	12.639	−968.58	0.984	0.015	0	−970.56	3.95	0.1384	0.9688
FGF17	645	1	0	0	0.019	1	−993.48	1	1E-005	0.019	−993.48	0.00	0.9997	1
FGF18	615	1	0	0	0.014	13.185	−865.28	1	1E-005	0.014	−865.28	0.00	0.9999	1
FGF19	525	1	0	0	0.038	1	−919.46	1	1E-005	0.038	−919.46	0.00	0.9998	1
FGF20	630	1	0	0	0.023	1	−949.80	1	1E-005	0.023	−949.80	0.00	0.9999	1
FGF21	600	1	0	0	0.207	1	−1161.23	1	1E-005	0.207	−1161.23	0.00	0.9999	1
FGF23	576	0.939	0.025	0.034	0.041	1	−961.53	0.939	0.060	0.041	−961.53	0	1	1

The results of the site model for each FGF genes. M2 is a selection model that allows local *d*_N_/*d*_S_ ratio to be higher than 1. *p*_0_, *p*_1_, and *p*_2_ are the proportions of codons with *d*_N_/*d*_S_ lower than 1, equals to 1, and higher than 1, respectively, and ω_0_ and ω_2_ are the average *d*_N_/*d*_S_ ratio of codons with *d*_N_/*d*_S_ < 1 and *d*_N_/*d*_S_ > 1, respectively. M1 is a neutral model that does not allow local *d*_N_/*d*_S_ ratio to be higher than 1. In this model, *p*_0_ and *p*_1_ are the pro*p*ortions of codons with *d*_N_/*d*_S_ lower than 1 an*d d*_N_/*d*_S_ equal to 1, respectively and ω_0_ is the average *d*_N_/*d*_S_ ratio of codons with *d*_N_/*d*_S_ lower than 1. The p values are calculated using likelihood ratio test (degree of freedom = 2) and further adjusted by the false discovery rate.

**Table 2:** Functional effect of ancestral cetacean mutations.

Genes	Accession number	Position	From	To	PolyPhen-2	SIFT	PROVEAN
FGF3	NP_005238.1	102	R	K	benign	Tolerated	Neutral
FGF5	NP_004455.2	54	M	K	benign	Tolerated	Neutral
FGF9	NP_002001.1	17	V	A	benign	Tolerated	Neutral
FGF9	NP_002001.1	27	V	A	benign	Tolerated	Neutral
FGF9	NP_002001.1	203	D	G	Probably damaging	Damaging	Deleterious
FGF10	NP_004456.1	43	D	G	benign	Damaging	Neutral
FGF11	NP_004103.1	63	G	C	Probably damaging	Tolerated	Neutral
FGF14	NP_787125.1	197	P	A	benign	Tolerated	Neutral
FGF19	NP_005108.1	113	L	S	Possibly damaging	Tolerated	Neutral
FGF19	NP_005108.1	177	E	D	benign	Tolerated	Neutral
FGF20	NP_062825.1	46	R	L	benign	Damaging	Neutral
FGF21	NP_061986.1	107	D	E	benign	Tolerated	Neutral
FGF21	NP_061986.1	119	E	Q	benign	Damaging	Neutral

List of amino acid changes that were generated in the ancestral cetacean. The name of genes, accession number, position in the alignment, and the functional prediction using PolyPhen-2, SIFT, and PROVEAN are shown.

**Table 3:** The comparison of amino acid substitution rate.

Method	Ancestral whale				The rest					
	The number of substitution		The rate of substitution		The number of substitution		The rate of substitution		Ratio (Anc Whale/Rest)	
	Damaging	Neutral	Damaging	Neutral	Damaging	Neutral	Damaging	Neutral	Damaging	Neutral
Poly Phen-2	3	10	0.077	0.256	681	5720	0.029	0.247	2.619	1.038
SIFT	4	9	0.103	0.231	808	5644	0.035	0.243	2.943	0.948
PROVEAN	1	12	0.026	0.308	414	6038	0.018	0.260	1.436	1.183

The Number of predicted damaging and neutral substitution by PolyPhen-2, SIFT, and PROVEAN in the ancestral branches of whales and the rest of the branches. The rate of substitution is calculated from the number of amino acid changes divided by the number of DNA substitution at third codon positions.
