# Supplementary material for: Analysis of the FGF gene family provides insights into aquatic adaptation in cetaceans
Source: Sci Rep. 2017 Jan 11;7:40233. doi: 10.1038/srep40233 (PMC5225608; doi:10.1038/srep40233)
Supplement: Supplementary Information [file srep40233-s1.pdf]

Supplementary Information

Analysis of the FGF gene family provides insights into aquatic adaptation in cetaceans

Kiwoong Nam[1,2,†], Kyeong Won Lee[3†], Oksung Chung[4†], Hyung-Soon Yim[3,5], Sun-Shin Cha[6], Sae-Won Lee[7], JeHoon Jun[4], Yun Sung Cho[4,8,9], Jong Bhak[4,8,9], Joao Pedro de Magalhaes[10], Jung-Hyun Lee[3,5*], Jae-Yeon Jeong[3,5,*]

This file includes

Supplementary Table S1. The Branch model.
Supplementary Table S2. The Branch-site model
Supplementary Table S3. RNA-seq analysis of FGF expression in tissues derived from a minke whale
Supplementary Table S4. Analysis of FGF expression in tissues derived from bowhead whales.

Supplementary Figure S1. A ML tree of FGF genes.
Supplementary Figure S2. Multiple amino acid sequence alignment of FGF3.
Supplementary Figure S3. Multiple nucleotide sequence alignment of FGF22 exons of the cetaceans and African elephant.
Supplementary Figure S4. Multiple amino acid sequence alignment of cetacean and African elephant FGF22.
Supplementary Figure S5. Positive selection on each codon.
Supplementary Figure S6. MEME analysis.

Supplementary Tables S5-S9 are provided as an excel file online.

Supplementary Table S5. Sequence data and cDNA assembly methods used in this study
Supplementary Table S6. Accession numbers and gene assembly method for FGF22.
Supplementary Table S7. Accession numbers of FGF11 promoters.
Supplementary Table S8. Accession numbers of FGF23 promoters.
Supplementary Table S9. Primers used for qPCR.

Gene	Number of sites		Two ratio model			One ratio model		p value	Adjusted p value
	N	S	d_N/d_S whales	d_N/d_S other mammals	Likelihood	d_N/d_S	Likelihood		
FGF1	351.8	107.2	0.0568	0.0641	-2473.60642	0.0638	-2473.62464	0.98194596	0.98194596
FGF2	333.5	119.5	0.0703	0.0526	-1600.78085	0.0536	-1600.84201	0.94067178	0.98194596
FGF3	405.1	74.9	0.2303	0.0539	-2589.11528	0.0681	-2599.5576	2.92E-005	0.00030631
FGF4	513.6	74.4	0.0383	0.0563	-2790.70149	0.0544	-2791.13462	0.64847943	0.98194596
FGF5	555.8	194.2	0.2858	0.1561	-4453.5157	0.1625	-4455.26673	0.17359522	0.72909993
FGF6	480.5	137.5	0.1267	0.0956	-3489.97433	0.0981	-3490.2961	0.72486634	0.98194596
FGF7	428.6	150.4	0.0494	0.0905	-2235.77206	0.0894	-2235.94878	0.83801188	0.98194596
FGF8	345.1	62.9	1.00E-004	0.0074	-1555.29087	0.0068	-1556.06016	0.46334053	0.98194596
FGF9	461.1	162.9	0.127	0.013	-2255.68795	0.0171	-2260.11688	0.01192722	0.08281435
FGF10	447.4	140.6	0.0984	0.0449	-2362.32127	0.0478	-2363.2451	0.39699365	0.98194596
FGF11	458.8	126.2	0.6242	0.0303	-2335.55845	0.0484	-2358.91474	0	[a]0
FGF12	525.1	194.9	1.00E-004	0.0081	-2325.57971	0.0077	-2325.87046	0.74770632	0.98194596
FGF13	492.8	182.2	1.00E-004	0.0066	-1735.83662	0.0061	-1736.07873	0.78496983	0.98194596
FGF14	549.6	191.4	0.0344	0.0279	-2029.12563	0.0286	-2029.15822	0.96793727	0.98194596
FGF16	475.9	145.1	0.0259	0.0433	-2452.1283	0.0419	-2452.38833	0.77103462	0.98194596
FGF17	540.1	104.9	0.0198	0.0159	-2589.54557	0.0161	-2589.60262	0.94454401	0.98194596
FGF18	516	99	0.014	0.0109	-1880.43876	0.0111	-1880.46357	0.97549817	0.98194596
FGF19	431.6	93.4	0.0391	0.1282	-4061.71248	0.1205	-4065.86186	0.01577416	0.08281435
FGF20	453.7	176.3	0.0262	0.0615	-2483.68263	0.0598	-2484.10561	0.65508846	0.98194596
FGF21	435.1	164.9	0.1987	0.1855	-4728.1053	0.1864	-4728.13531	0.97044165	0.98194596
FGF23	484	95	0.1021	0.1181	-4287.68182	0.1172	-4287.76756	0.91783008	0.98194596

Supplementary Table S1. The Branch model. The names of FGF genes, numbers of nonsynonymous and synomynous sites, the average d_N/d_S ratios of the whale branches and those of the other branches, the likelihoods of two ratio model that allows different d_N/d_S ratios between whale branches and the other branches, the likelihoods of one ratio model that allows only single d_N/d_S ratio across the mammalian phylogenetic tree, p values calculated from log likelihood ratio test, and the FDR adjusted p values are also shown. a: $< 2.2 \times 10^{-16}$.

Gene	Selection model						Neutral model					2ΔL
	p_0	p_1	p_2	ω_0	ω_2	L	p_0	p_1	p_2	ω_0	L	
FGF1	0.96981	0.03019	0	0.04378	1	-2440.06	0.96981	0.03019	0	0.04378	-2440.06	0
FGF2	0.88589	0.03024	0.08387	0.03317	2.25932	-1595.31	0.87678	0.02993	0.09329	0.03317	-1595.31	-1.00E-005
FGF3	0.88943	0.11057	0	0.0326	1	-2531.59	0.88943	0.11057	0	0.0326	-2531.59	0
FGF4	0.9609	0.0391	0	0.04568	1	-2777.69	0.9609	0.0391	0	0.04568	-2777.69	0
FGF5	0.82581	0.16069	0.0135	0.05365	1.55487	-4341.39	0.8209	0.15974	0.01936	0.05366	-4341.39	0.005882
FGF6	0.92132	0.05793	0.02075	0.06726	5.75385	-3456.15	0.84982	0.05317	0.09701	0.06736	-3456.19	0.072764
FGF7	0.93828	0.06172	0	0.0507	1	-2214.42	0.93828	0.06172	0	0.0507	-2214.42	0
FGF8	1	0	0	0.00683	1	-1556.06	1	0	0	0.00683	-1556.06	2.00E-006
FGF9	0.77165	0	0.22835	0.01387	1	-2255.81	0.77165	0	0.22835	0.01387	-2255.81	0
FGF10	0.98496	0	0.01504	0.04757	1	-2363.22	0.98496	0	0.01504	0.04757	-2363.22	0
FGF11	0.36704	0.01128	0.62168	0.03027	1	-2348.61	0.36704	0.01128	0.62168	0.03027	-2348.61	0
FGF12	0.98999	0.01001	0	0.00231	1	-2321.56	0.98999	0.01001	0	0.00231	-2321.56	0
FGF13	1	0	0	0.00607	1	-1736.07	1	0	0	0.00607	-1736.07	0
FGF14	0.9588	0.01508	0.02612	0.01525	1	-2023.41	0.9588	0.01508	0.02612	0.01525	-2023.41	0
FGF16	0.9764	0.0236	0	0.02846	1	-2441.85	0.9764	0.0236	0	0.02846	-2441.85	0
FGF17	1	0	0	0.01612	1	-2589.60	1	0	0	0.01612	-2589.60	0
FGF18	0.97841	0.02159	0	0.00284	1	-1858.25	0.97841	0.02159	0	0.00284	-1858.25	0
FGF19	0.91387	0.08613	0	0.09341	1	-4010.11	0.91387	0.08613	0	0.09341	-4010.11	0
FGF20	0.9694	0.02499	0.00561	0.04509	20.0918	-2479.59	0.9043	0.02336	0.07234	0.04508	-2479.60	0.03544
FGF21	0.72987	0.21516	0.05497	0.07573	1	-4622.97	0.72987	0.21516	0.05497	0.07573	-4622.97	0
FGF23	0.83993	0.16007	0	0.07071	1	-4199.59	0.83993	0.16007	0	0.07071	-4199.59	0

Supplementary Table S2. The branch site model. The name of FGF genes, the estimated parameters, and the two times of difference in the likelihoods (L) are shown. For the selection model, in the foreground branches (the whale branches) p_0, p_1, and p_2 are the proportions of codons with d_N/d_S lower than 1, equals to 1, and higher than 1, respectively, and ω_0 and ω_2 are the average d_N/d_S ratio of codons with d_N/d_S <1 and d_N/d_S >1, respectively. For the neutral model, ω_2 is fixed to be 1, thus no codon is allowed to have d_N/d_S higher than 1. Significance of positive selection was tested by the comparison of likelihoods between selection model and neutral models. If two times of the difference in likelihood is not larger than 3.84, we regarded that positive selection is not supported. See the Method.

Gene	Brain	Heart	Lung	Kidney	Liver	Muscle
FGF1	27.00	135.04	3.53	31.46	6.08	3.59
FGF2	5.64	0.00	2.67	1.17	0.00	0.00
FGF5	10.93	0.00	0.00	0.00	0.00	0.00
FGF6	4.17	0.00	0.00	0.00	0.00	0.00
FGF7	0.00	0.00	69.79	1.13	3.06	3.58
FGF8	0.00	0.00	0.00	0.00	0.00	0.00
FGF9	20.95	0.00	0.00	26.78	0.00	0.00
FGF10	0.00	0.00	0.00	0.00	0.00	0.00
FGF11	**159.47**	**280.82**	2.64	11.84	18.17	11.96
FGF12	49.88	0.00	2.64	0.51	0.00	0.00
FGF13	15.43	10.02	0.85	0.51	9.11	19.70
FGF14	25.56	1.57	0.00	0.51	1.55	0.00
FGF17	1.48	0.00	0.00	0.00	0.00	0.00
FGF19	0.00	0.00	0.00	0.00	0.00	0.00
FGF20	0.00	0.00	0.00	0.00	1.55	0.00
FGF21	0.40	0.00	0.00	0.00	3.06	0.00
FGF23*	0.00	0.00	0.00	0.00	**110.17**	0.00

Supplementary Table S3. RNA-seq analysis of FGF expression in tissues derived from a minke whale[23]. Values represent read counts normalized by Trimmed Mean of M-values (TMM) using the edgeR. *The expression level of *FGF23* was calculated by transcriptome mapping to bowhead whale genome sequence because *FGF23* gene region in the minke whale reference genome sequence was incompletely assembled.

Whale #	Brain<br>1	Heart<br>1	Kidney<br>1	 2	 3	 4	 5	Liver<br>1	 3	 4	 5	Muscle<br>1
FGF1	28.44	68.81	229.90	30.48	60.14	40.85	127.37	11.74	8.30	16.77	21.46	57.41
FGF2	25.64	5.06	7.07	3.95	3.38	3.70	4.70	13.30	8.28	11.36	19.91	5.06
FGF6	13.00	0. 00	0.00	0.00	0.00	0.00	2.37	0.00	0.00	0.00	0.00	4.88
FGF7	0.90	24.27	14.27	0.00	1.95	0.00	2.21	4.99	7.85	3.88	1.75	40.49
FGF8	0.00	0.00	0.00	0.00	0.00	0.00	0.00	0.00	0.00	0.00	0.00	0.00
FGF9	29.29	4.05	41.36	21.70	25.91	25.02	33.26	0.09	0.09	1.82	2.88	7.60
FGF10	0.00	0.00	3.79	0.00	2.17	3.17	0.50	1.46	0.04	0.05	0.03	0.04
FGF11	0.56	0.00	0.00	2.62	0.00	0.00	0.00	0.00	0.00	0.00	0.00	0.02
FGF12	62.11	0.00	7.00	3.12	4.06	6.74	2.81	0.00	0.00	0.00	0.00	1.37
FGF13	18.16	5.06	5.56	0.00	0.55	0.64	2.81	4.64	0.12	1.97	1.64	61.32
FGF14	41.62	0.00	4.18	0.00	0.56	1.41	0.42	1.57	0.07	0.09	1.49	0.08
FGF16	0.00	7.07	4.31	4.88	1.30	0.67	3.63	0.00	0.00	0.00	0.00	2.53
FGF17	0.00	0.00	0.00	0.00	0.78	0.00	0.00	0.00	0.00	0.00	0.00	0.00
FGF19	0.00	0.00	0.00	0.00	0.00	0.00	0.00	0.00	0.00	0.00	0.00	0.00
FGF20	8.66	0.00	0.00	0.00	0.00	0.00	0.00	0.00	0.00	0.00	0.00	0.04
FGF21	0.00	0.00	0.00	0.00	0.00	0.00	0.00	0.09	1.61	0.12	2.88	0.00
FGF23	2.10	0.00	0.00	0.00	0.00	0.00	0.00	**0.25**	**1.91**	**23.71**	**24.34**	0.01

Supplementary Table S4. Analysis of FGF expression in tissues derived from bowhead whale. The expression levels of each FGF were analyzed using RNA-seq data from Keane M et al. (whale 1)[22] and Seim I et al. (whales 2-5)[35]. Tissues from 2 - 5 are derived from individuals as follows: 2, 10B15; 3, 10B16; 4, 10B18; 5, 10B21. Values represent read counts normalized by Trimmed Mean of M-values (TMM) using the edgeR.

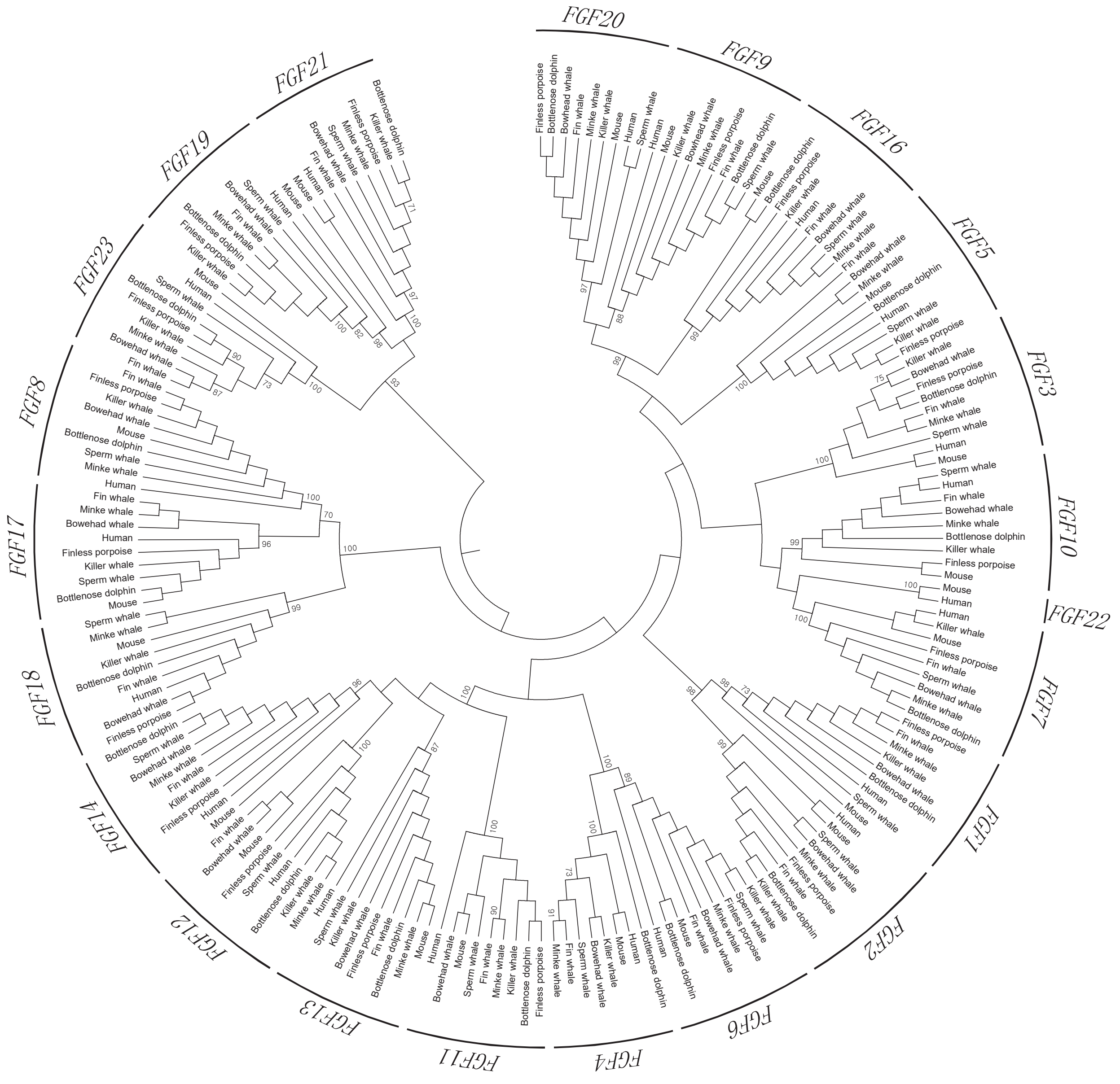

Supplementary Figure S1. A ML tree of FGF genes. Topology of the ML tree is based on the coding sequences of all of the FGF genes generated by combining our newly identified FGF sequences from cetaceans with those from human and mouse. Numbers along the branches are bootstrap support values. Values below 70 are not shown.

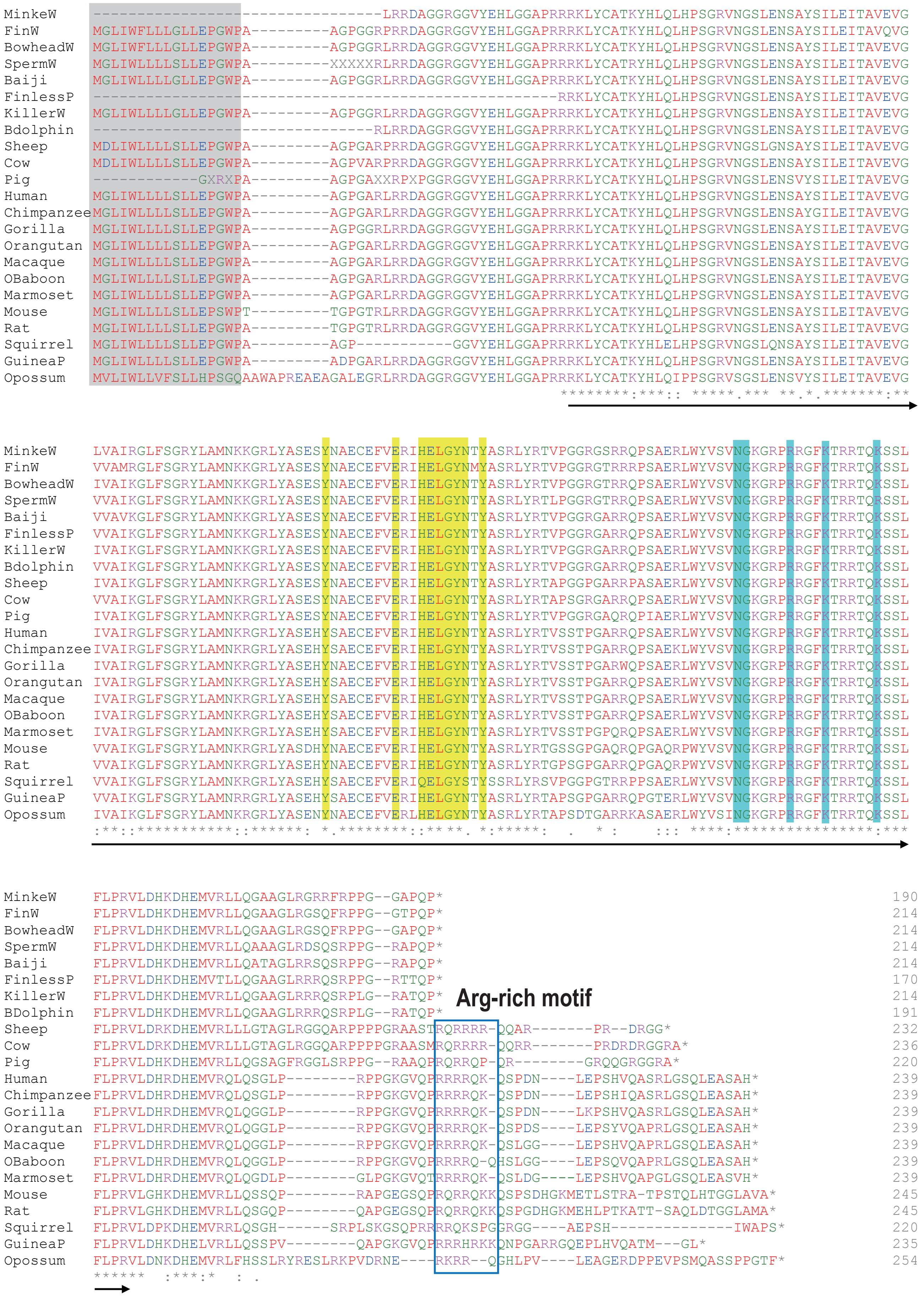

Supplementary Figure S2. Multiple amino acid sequence alignment of FGF3. Signal sequence, receptor binding sites, and heparin-binding sites are highlighted in grey, yellow, and blue, respectively. FGF domain is underlined with arrow, and C-terminal Arg-rich sequence is marked with blue square.

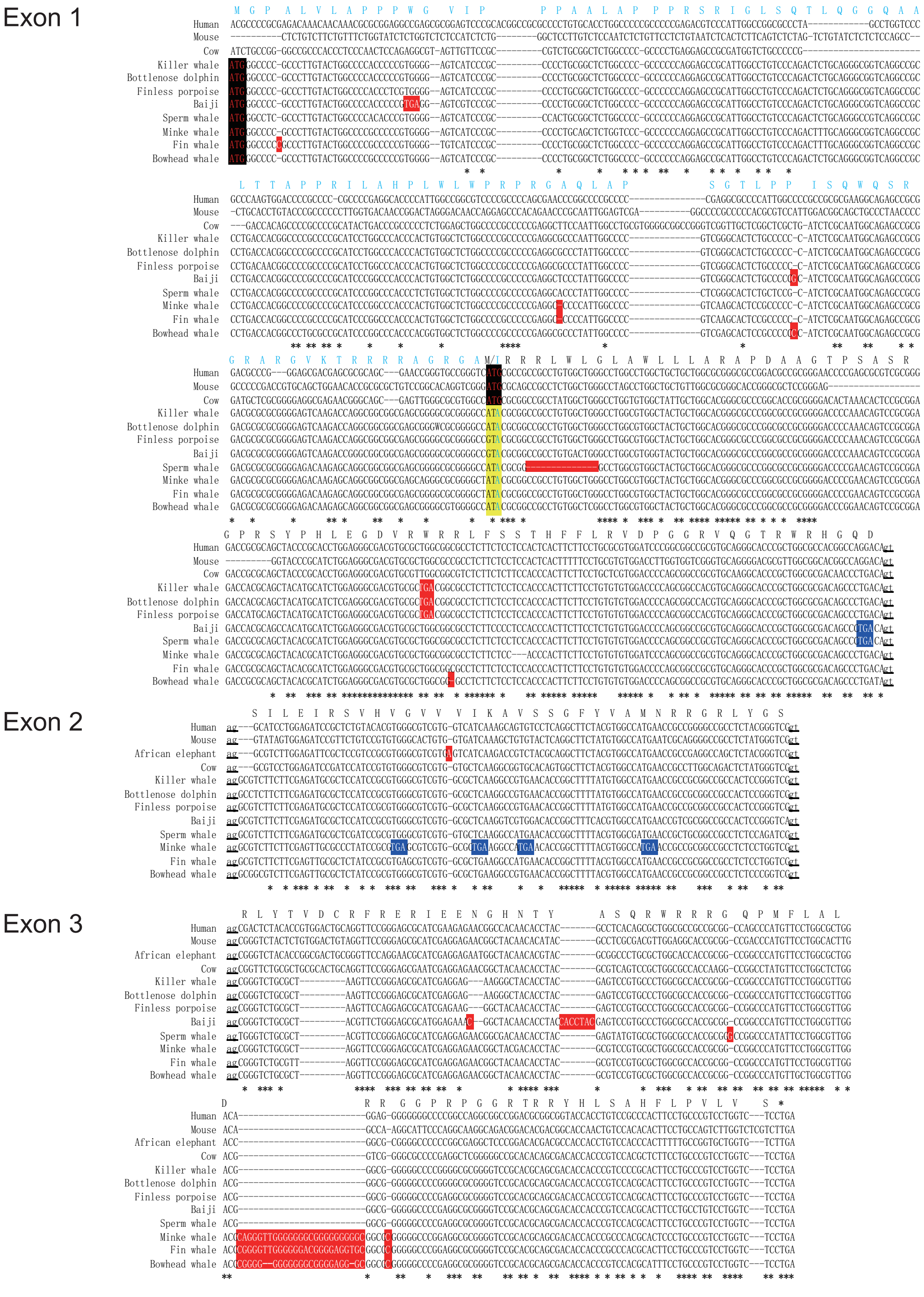

Figure S3 Multiple nucleotide sequence alignment of *FGF22* exons of the cetaceans and African elephant. For comparison, Human, mouse, and cow sequences are included in the alignment. Start codons are marked with black boxes. The G to A substitution in the canonical start codon that caused cetacean-specific N-terminal extension is marked in blue with a yellow box. Amino acids in blue and black represent killer whale and human FGF22 sequences, respectively. Damaging mutations are marked with red boxes, and premature stop codons due to frameshift mutations were shown with blue boxes. Exon 1 of the African elephant *FGF22* was deleted and was not shown in this figure.

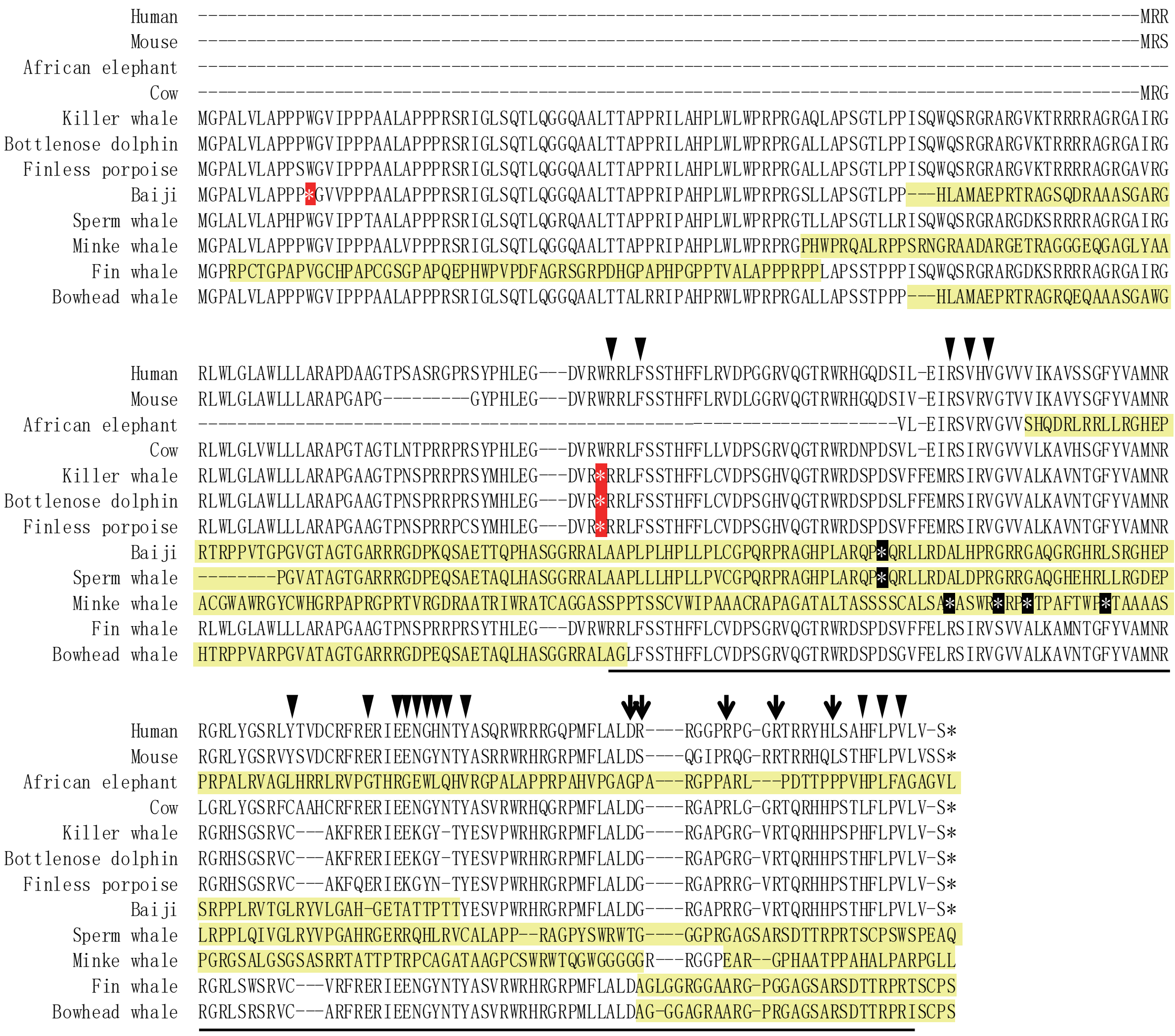

Supplementary Figure S4. Multiple amino acid sequence alignment of cetacean and African elephant FGF22. For comparison, Human, mouse, and cow sequences are included in the alignment. Premature stop codons due to a single nucleotide substitution are marked with asterisks in red boxes, and premature stop codons due to frameshift mutations are marked with asterisks in black boxes. Frameshift mutations are highlighted in yellow. Arrowheads and arrows indicate potential receptor-, and heparin-binding sites, respectively. FGF domain is underlined.

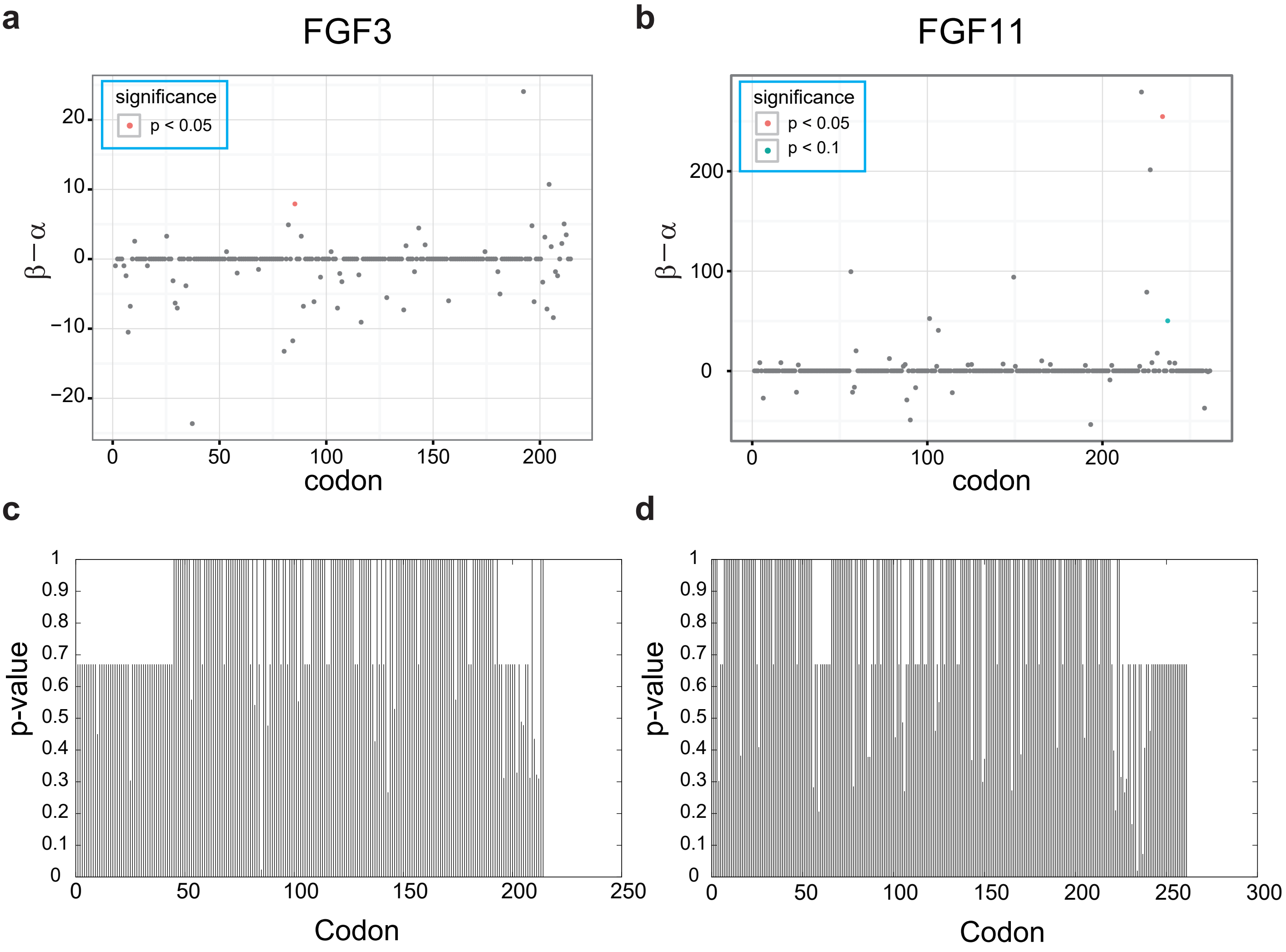

Supplementary Figure S5. Positive selection on each codon. (**a, b**) The plots show the difference between non-synonymous substitution rate (β) and synonymous substitution rate (α) at each codon of FGF3 (a) and FGF11 (b), based on the MEME software. The difference between α and β is tested using likelihood ratio test and codons with p-values lower than 0.05 and 0.1 are indicated by pink and turquoise colors, respectively. (**c, d**) Manhattan plots showing the distribution of p-values indicate the significance level of positive selection on each codon of FGF3 (c) and FGF11 (d), based on the MEME software.

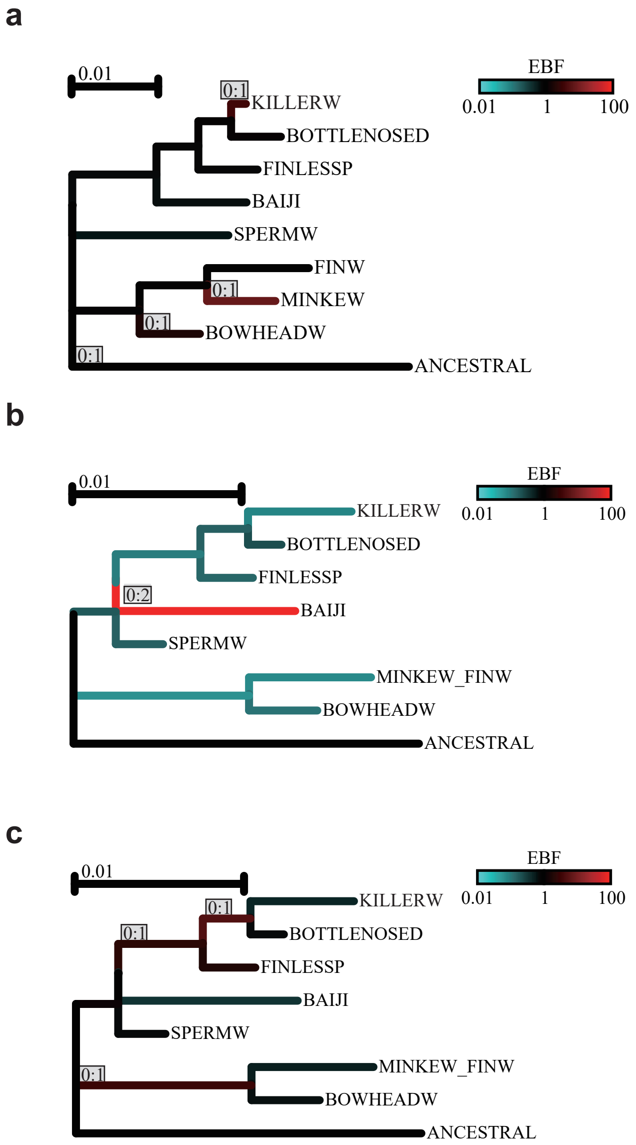

Supplementary Figure S6. MEME analysis. Empirical Bayes factors representing the likelihood of positive selection at (**a**) 85th residue of FGF3, (**b**) 234th residue of FGF11, and (**c**) 237th residue of FGF11 are shown on each branch of whale phylogenetic trees. The first and second digits in the grey squares represent the number of synonymous and nonsynonymous substitutions, respectively.
